# Supplementary material for: SNM1A is crucial for efficient repair of complex DNA breaks in human cells
Source: Nat Commun. 2024 Jun 25;15:5392. doi: 10.1038/s41467-024-49583-5 (PMC11199599; doi:10.1038/s41467-024-49583-5)
Supplement: Supplementary file 1 — Supplementary Information [file 41467_2024_49583_MOESM1_ESM.pdf]

(1) **TCATAAACTGGACGTATCTTTGG** [posn: 334 - 356, -ve]

| target genomic coordinates | strand | genome seq | RefSeq transcript |
|---|---|---|---|
| chr10:115612586-115612608 | +ve | TCATAAACTGGACGTATCTTTGG | NM_001271816, NM_014881 |

(2) **ACATTTCCGAAGAAGACATTTGG** [posn: 11 - 33, +ve]

| target genomic coordinates | strand | genome seq | RefSeq transcript |
|---|---|---|---|
| chr10:115612909-115612931 | -ve | ACATTTCCGAAGAAGACATTTGG | NM_001271816, NM_014881 |

**Supplementary Table 1** *sgRNA used in this study for deletion of the majority of exon 1 in DCLRE1A*

| Antibody | Supplier | Cat #(Clone#) | Lot Number | Species | Clonality | Dilution used[1] |
|---|---|---|---|---|---|---|
| Glutathione-S-Transferase | Merck | G1160 | 087H4806 | Mouse | Mono | 1:1000 |
| APLF | Abcam | AB105446 | GR119254-1 | Rabbit | Poly | 1:1000 |
| Anti-poly-ADP-ribose | R&D systems | 4335-MC-100 | 20554 | Mouse | Mono | 1:1,000 |
| SNM1A | Bethyl Laboratories | A303-747A | 1 | Rabbit | Poly | 1:1000 |
| Ub PCNA (K164) | Cell Signalling | 134395 [D5C7P] | 4 | Rabbit | Mono | 1:1000 |
| PCNA (Ms) | Santa Cruz | SC-056 [PC-10] | C2922 | Mouse | Mono | 1 :1000 |
| PCNA (Rb) | Abcam | AB92552 [EPR3821] | GR244165-29 | Rabbit | Mono | |
| BrdU | Abcam | AB6326 [BU1/75 (ICR1)] | GR267766-1 | Rat | Mono | 1:1000 |
| HA.11 | BioLegend | 901533 [16B12] | B350093 | Mouse | Mono | |
| β-Actin-HRP | Merck | A3854 [AC-15] | 239228 | Mouse | Mono | 1:20,000 |
| 53BP1 (Ms) | Millipore | MAB3802 [BP13] | 3173790 | Mouse | Mono | |
| 53BP1 (Rb) | Abcam | AB175933 [EPR2172(2)] | GR3225663-2 | Rabbit | Mono | |
| γH2AX (Ms) | Millipore | 05-636-I [JBW301] | 3308857 | Mouse | Mono | |
| γH2AX (Rb) | Abcam | Ab81299 [EP854(2)Y] | GR3203642-12 | Rabbit | Mono | |
| Goat anti-Rabbit-HRP | Dako | P0448 | 41424306 | Goat | Poly | 1:5,000 |
| Goat anti-Mouse-HRP | Dako | P0447 | 000776656 | Goat | Poly | 1:5,000 |
| Alexa Fluor 488 (anti-Rat) | Invitrogen | A11006 | 2416486 | Goat | | 1:500 |
| Alexa Fluor 488 (anti-Rabbit) | Invitrogen | A11008 | 712170 | Goat | | |
| Alexa Fluor 555 (anti-Rabbit) | Invitrogen | A31572 | 5294873 | Donkey | | |
| Alexa Fluor 647 (anti-Rabbit) | Invitrogen | A21443 | 1917946 | Chicken | | |
| Alexa Fluor 555 (anti-Mouse) | Invitrogen | A31570 | 1575605 | Donkey | | |
| Alexa Fluor 647 (anti-Mouse) | Invitrogen | A31571 | 1839633 | Donkey | | |

[1] Dilutions listed are for western blot protocols. All dilutions for microscopy work were 1:200 for primary antibodies and 1:1,000 for secondary antibodies.

**Supplementary Table 2** *List of antibodies used in this study.*

| Gene | Forward sequence | Reverse sequence |
| --- | --- | --- |
| BRCA2 siRNAs: | GGGAAACACUCAGAUUAAAUUdTdT | AAUUUAAUCUGAGUGUUUCCCdTdT |
| RNF8 siRNAs: | GGACAAGAGCGAUGGAGGAdTdT | UUGUUGUCCAUAAUUGUCCdTdT |
| RNF168 siRNAs: | GGCGAAGAGCGAUGGAGGAdTdT | UCCUCCAUCGCUCUUCGCCdTdT |

**Supplementary Table 3** *List of siRNA sequences used in this study.*

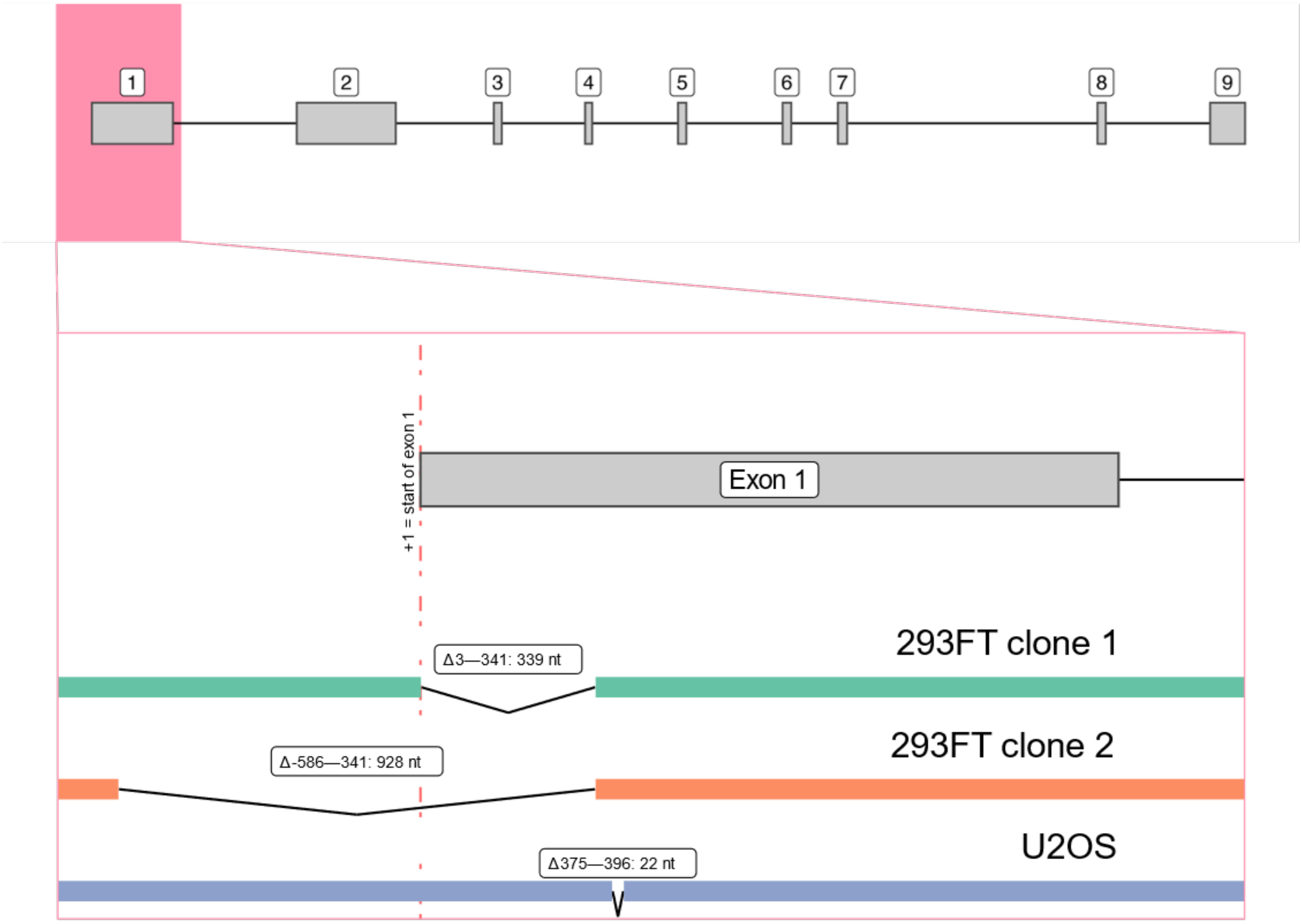


| Clone/Cell line | Deletion |
|---|---|
| U2OS | 375 to 396 |
| 293FT clone 1 | 3 to 341 |
| 293FT clone 2 | -586 to 341 |

**Supplementary Figure 1** *Description of deletions introduced in SNM1A gene in the cells used in this study.* Regions of SNM1A deleted in disrupted cell lines. Upper panel; schematic of exon-intron structure of SNM1A. Lower panels and table; expanded views and coordinates of the nucleotide deletions within exon 1 of the gene in the U2OS-derived disruptants and in the two 293FT clones employed.

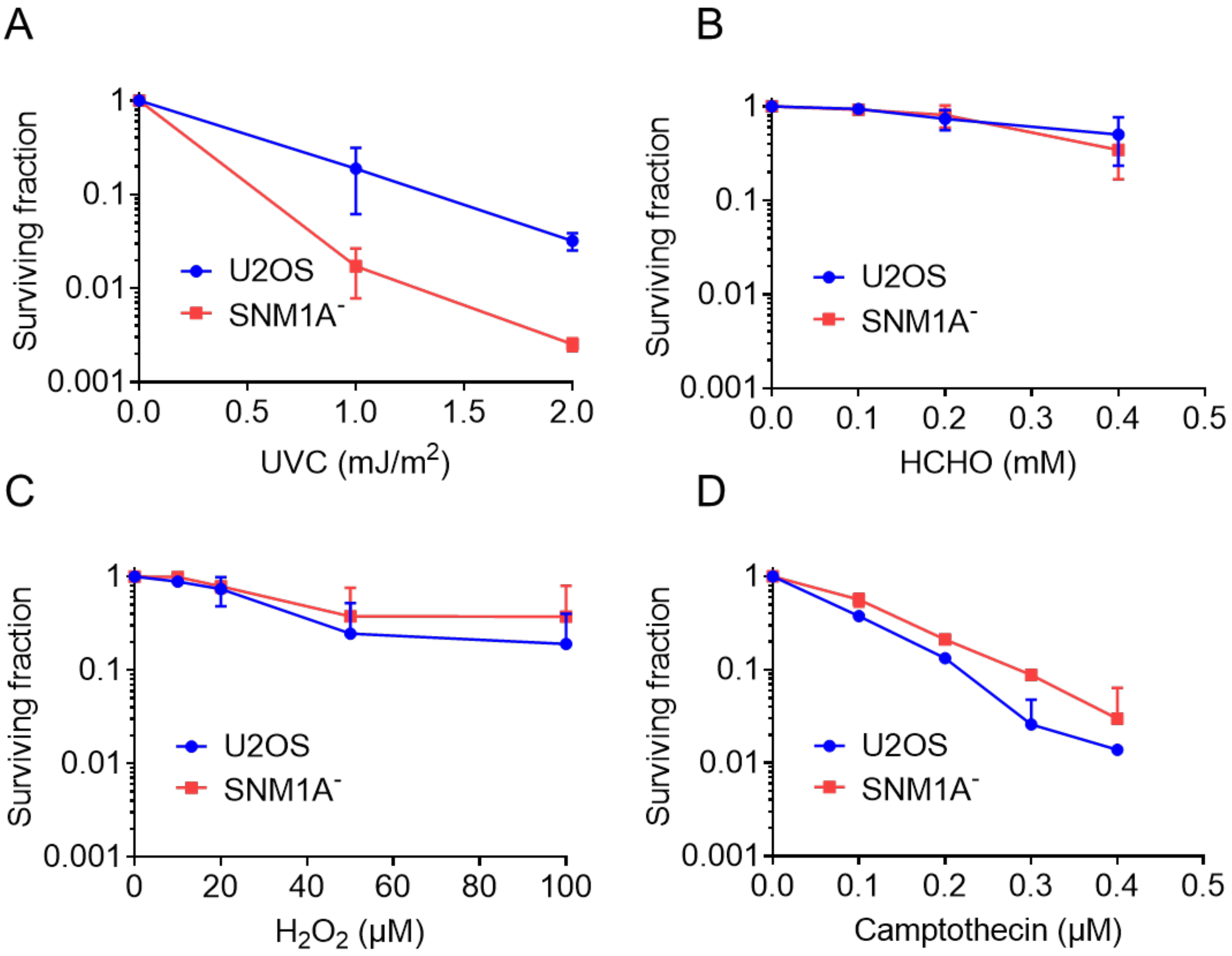


**Supplementary Figure 2** *Clonogenic survival assays in U2OS and SNM1A*$^{-}$ *cells.* **A.** Clonogenic survival assays were performed on U2OS and SNM1A$^{-}$ cells to treatments of UVC (254 nm). **B.** Formaldehyde (HCHO, 2 hours), **C.** hydrogen peroxide ($H_2O_2$, continuous) and **D.** Camptothecin (1 hour). Treated cells were allowed to grow for 12 days before being stained with Coomassie Brilliant Blue R250. Colonies were counted and the data plotted for biological repeats (A = 2, B = 6, C = 4, D = 2) containing duplicate plates per dose and normalised to the control (untreated). Error is the standard error of the mean (SEM).

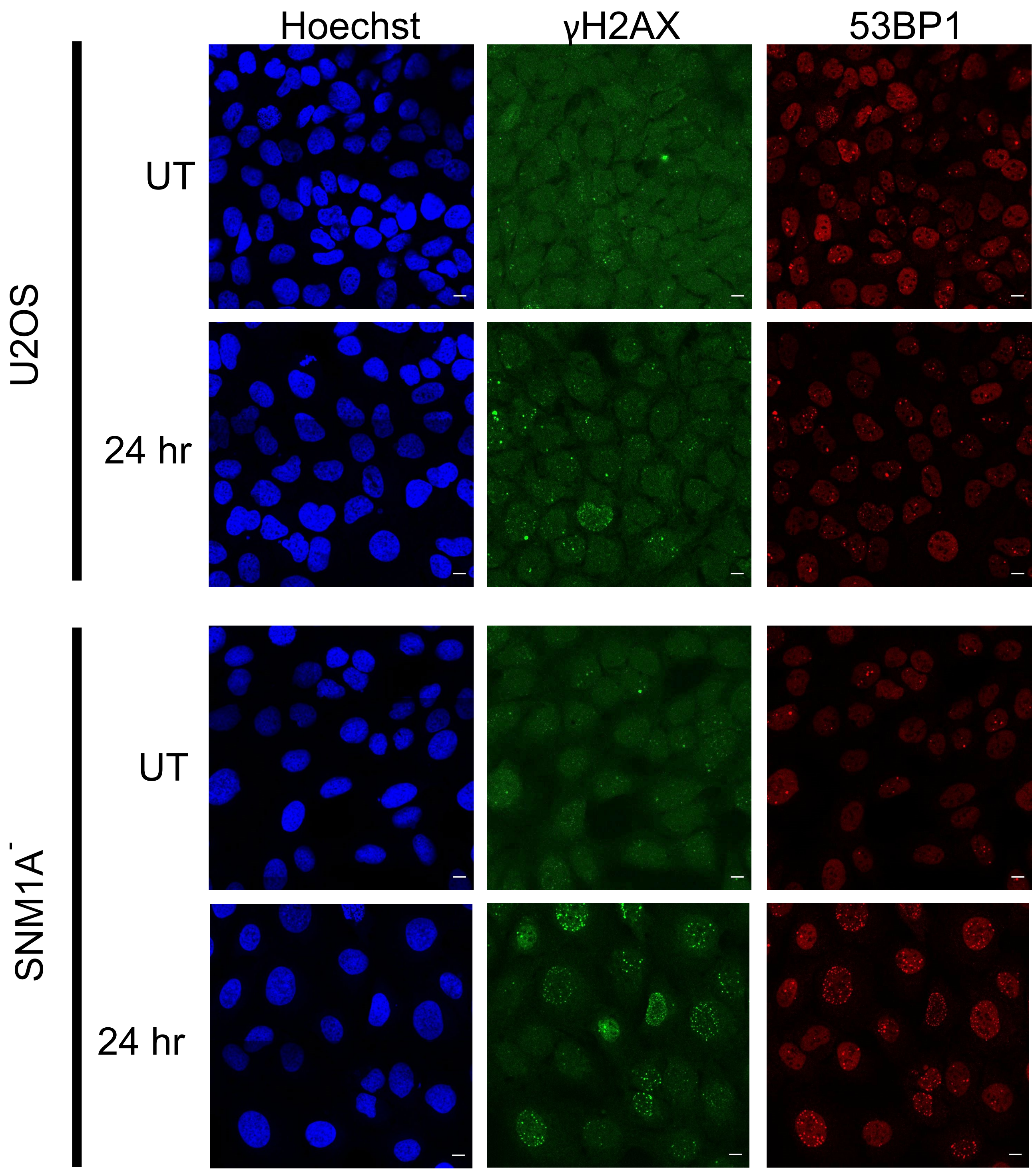


**Supplementary Figure 3** *Zeocin induced γH2AX and 53BP1 foci in WT U2OS and SNM1A⁻ cells.* Zeocin treated cells (0.1 mg/mL, 2 hours then further 24 hours in the absence of drug) versus untreated cells. Representative images of a wider field of view of data represented in **Figure 1I**. Scale bar = 10 µm.

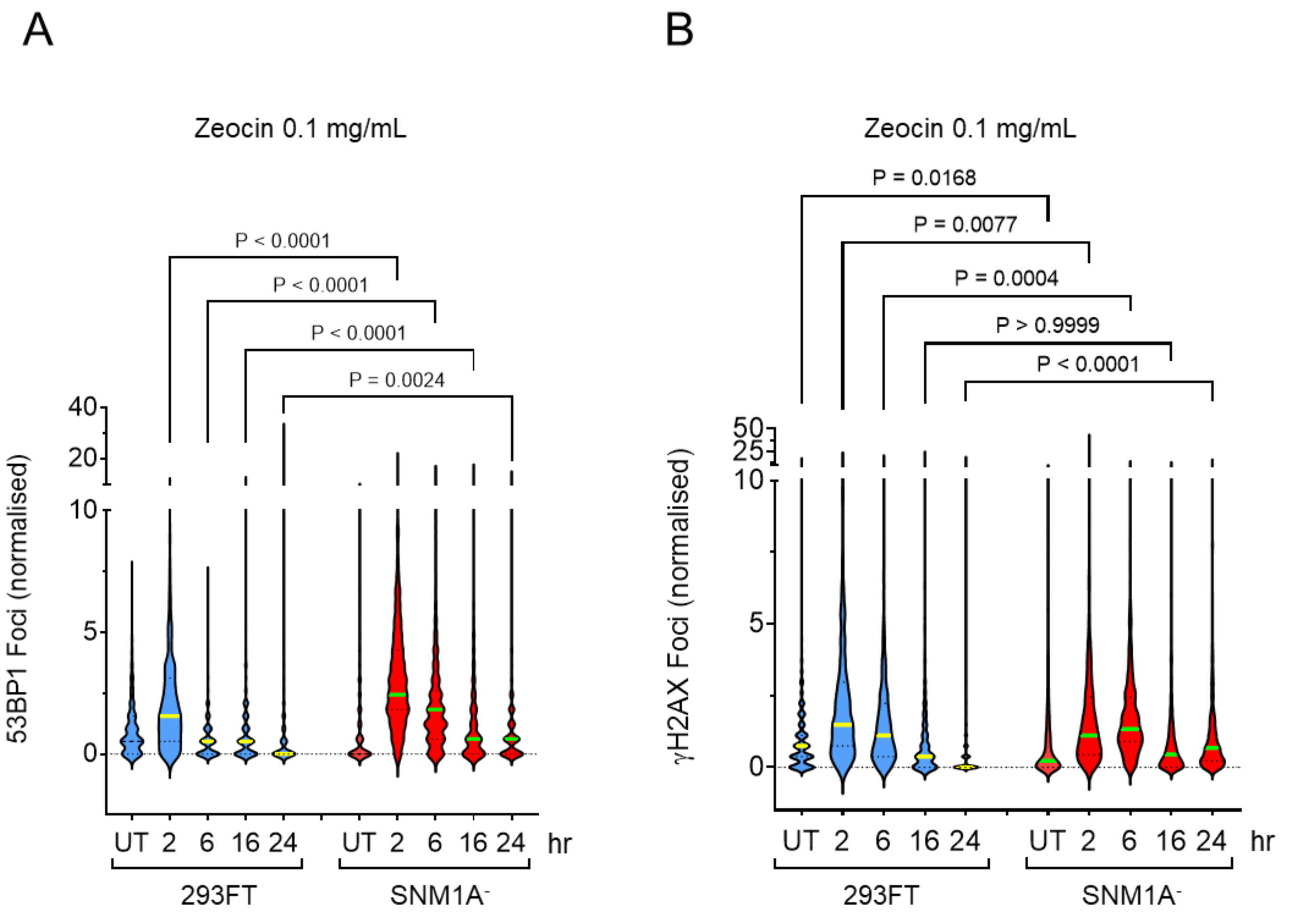


**Supplementary Figure 4** *Quantification of damage induced 53BP1 and γH2AX foci in 293FT and SNM1A⁻ cells* **A.** 53BP1 and **B.** γH2AX Foci in 293FT and SNM1A- cells following Zeocin treatment (0.1 mg/mL, 2 hr). The mean number of foci were compared between 293FT and SNM1A⁻ cells for each post-treatment timepoint. Data is from 3 biological repeats counting (from left to right) in **A:** 455, 506, 675, 546, 542, 588, 604, 670, 656, 663 cells and for **B**: 439, 413, 652, 565, 604, 623, 546, 568, 620, 629 cells. P values calculated with Kruskal-Wallis test (post-hoc Dunn's multiple comparison test)

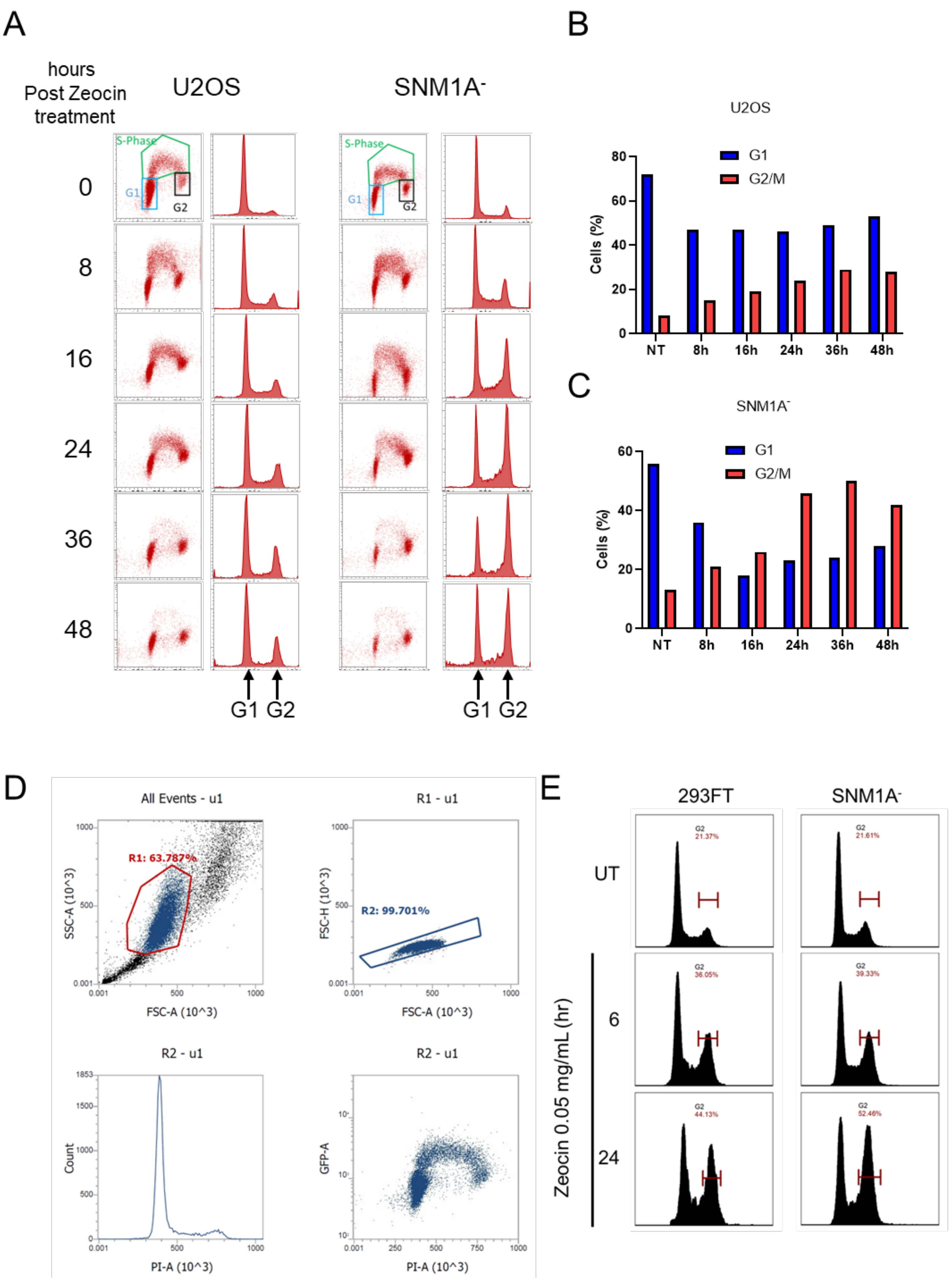

A
hours
Post Zeocin
treatment
U2OS
SNM1A-
S-Phase
G1
G2
0
8
16
24
36
48
G1 G2
G1 G2
B
U2OS
G1
G2/M
Cells (%)
NT
8h
16h
24h
36h
48h
C
SNM1A-
G1
G2/M
Cells (%)
NT
8h
16h
24h
36h
48h
D
All Events - u1
R1: 63.787%
SSC-A (10^3)
FSC-A (10^3)
R1 - u1
R2: 99.701%
FSC-H (10^3)
FSC-A (10^3)
R2 - u1
Count
PI-A (10^3)
R2 - u1
GFP-A
PI-A (10^3)
E
293FT
SNM1A-
UT
6
24
Zeocin 0.05 mg/mL (hr)
G2
21.37%
21.61%
36.05%
39.33%
44.13%
52.46%

**Supplementary Figure 5** *SNM1A$^{-}$ cells accumulate in G2/M phase of the cell cycle in response to Zeocin treatment.* Complete set of flow cytometry data as summarised in **Figure 1J**. **A.** U2OS and SNM1A$^{-}$ cells were treated with Zeocin (0.1 mg/mL continuously) and the cell cycle distribution analysed by BrdU incorporation. The accumulation of SNM1A$^{-}$ cells in G2/M-phase of the cell cycle can be seen from 16 hrs and this population persists through to 48 hrs. To enumerate this accumulation, the acquired data was gated to indicate G0/G1-phase cells (G1), S-phase and G2/M-phase (G2) populations and the percentage of cells in G1- compared with G2-phase of the cell cycle was plotted for **B.** U2OS and **C.** SNM1A$^{-}$ cells. The data for **A**, **B** and **C** is representative of 3 biological repeats. **D.** Example of gating scheme used in this study. Cells were analysed on a side scatter area versus forward scatter area profile and gated for viable events (region R1: top left panel). These viable cells were then gated forward scatter height versus forward scatter area (top right panel) to determine singlets over doublets. The singlets defined by the gated region R2 were then used to plot DNA content (labelled "PI-A", bottom left panel) and the BrdU incorporation (bottom right panel). BrdU incorporation was measured in the GFP channel with an Alexafluor-488 secondary to an anti-BrdU primary antibody (y axis being labelled "GFP-A") versus DNA content as above. **E.** Cell cycle distribution by DNA content determined by flow cytometry following Zeocin treatment (0.05 mg/mL, continuous) over 24 hours reveals greater G2 accumulation in SNM1A$^{-}$ cells over WT 293FT cells.

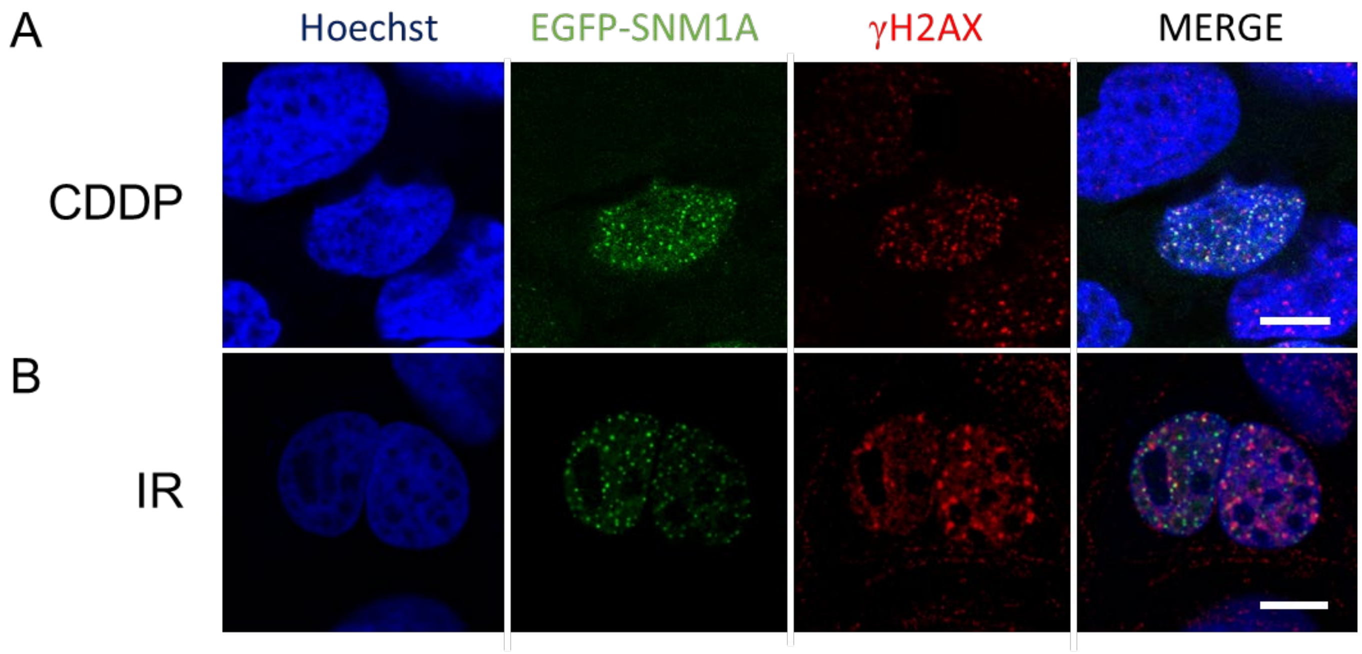


**Supplementary Figure 6** *Cisplatin and IR treatments induce EGFP-SNM1A and γH2AX foci in U2OS cells.* **A.** EGFP-SNM1A (green) and γH2AX (red) foci in response to Cisplatin (CDDP, 50 µM, 4 hour treatment followed by 24 hour recovery) and **B.** Ionising radiation (5 Gy, 2 hour) in U2OS EGFP-SNM1A stable cells. Cells were treated, fixed and probed with an antibody to γH2AX. Scale bar = 10 µm.

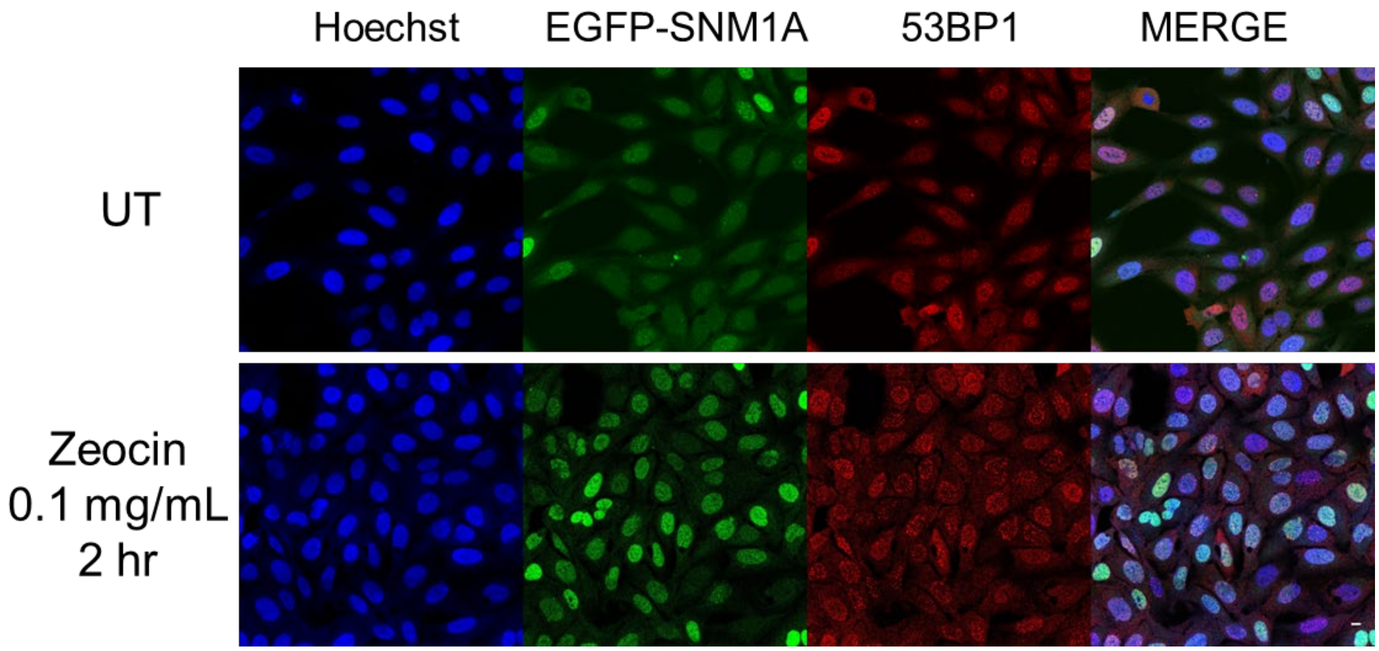


**Supplementary Figure 7** *Zeocin induced SNM1A and 53BP1 foci in U2OS cells.* Zeocin treated cells (0.1 mg/mL, 2 hours) versus untreated cells (UT). Representative images of a wider field of view of data represented in **Figure 2**. Scale bar = 10 μm.

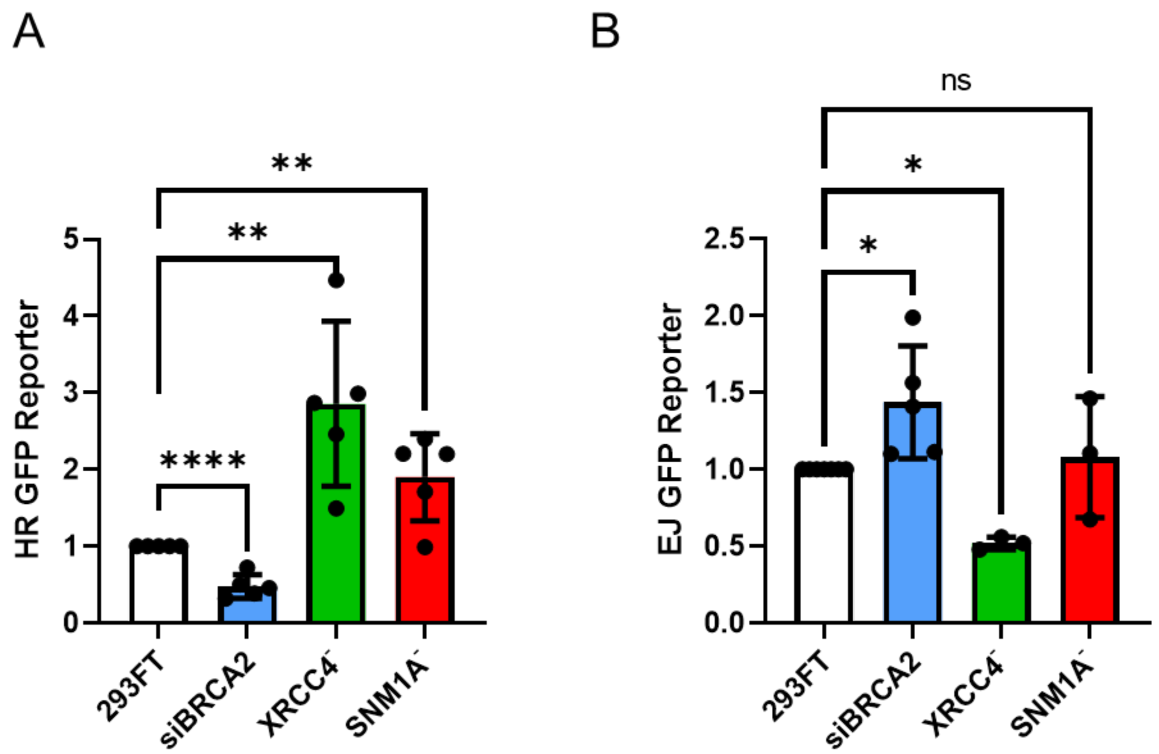


**Supplementary Figure 8** *SNM1A⁻ cells do not exhibit fundamental defects in homologous recombination or non-homologous end-joining.* Wild-type cells (293FT), along with cells deficient in homologous recombination repair (HR, treated with siRNA directed against BRCA2), cells deficient in non-homologous end-joining (NHEJ, genomic deletion of XRCC4) or SNM1A$^-$ cells were assayed for repair of double-strand breaks using I-SceI-reporter assays. Transfection of wild-type, siBRCA2, XRCC4$^-$ and SNM1A$^-$ cells with the HR reporter plasmid pDR-GFP **A.** or the NHEJ reporter plasmid pimEJ5GFP **B.** along with a plasmid containing the rare cutting enzyme I-SceI (pCMV-SceI). Co-transfected cells were incubated overnight, harvested, and analysed for GFP positive cells by flow cytometry. GFP positive cells for both these reporter assays indicate proficiency in repairing double-strand breaks (DSBs) induced by the co-transfected I-SceI containing plasmid. Data presented is the mean (error SEM) of five (all data in **A**, as well as 293FT and siBRCA2 in **B**) or three (XRCC4$^-$ and SNM1A$^-$ in **B**) biological repleats. Student's t test shows significance of the distribution where * = P≤0.05, ** = P≤0.01, *** = P≤0.001 and **** = P≤0.0001.

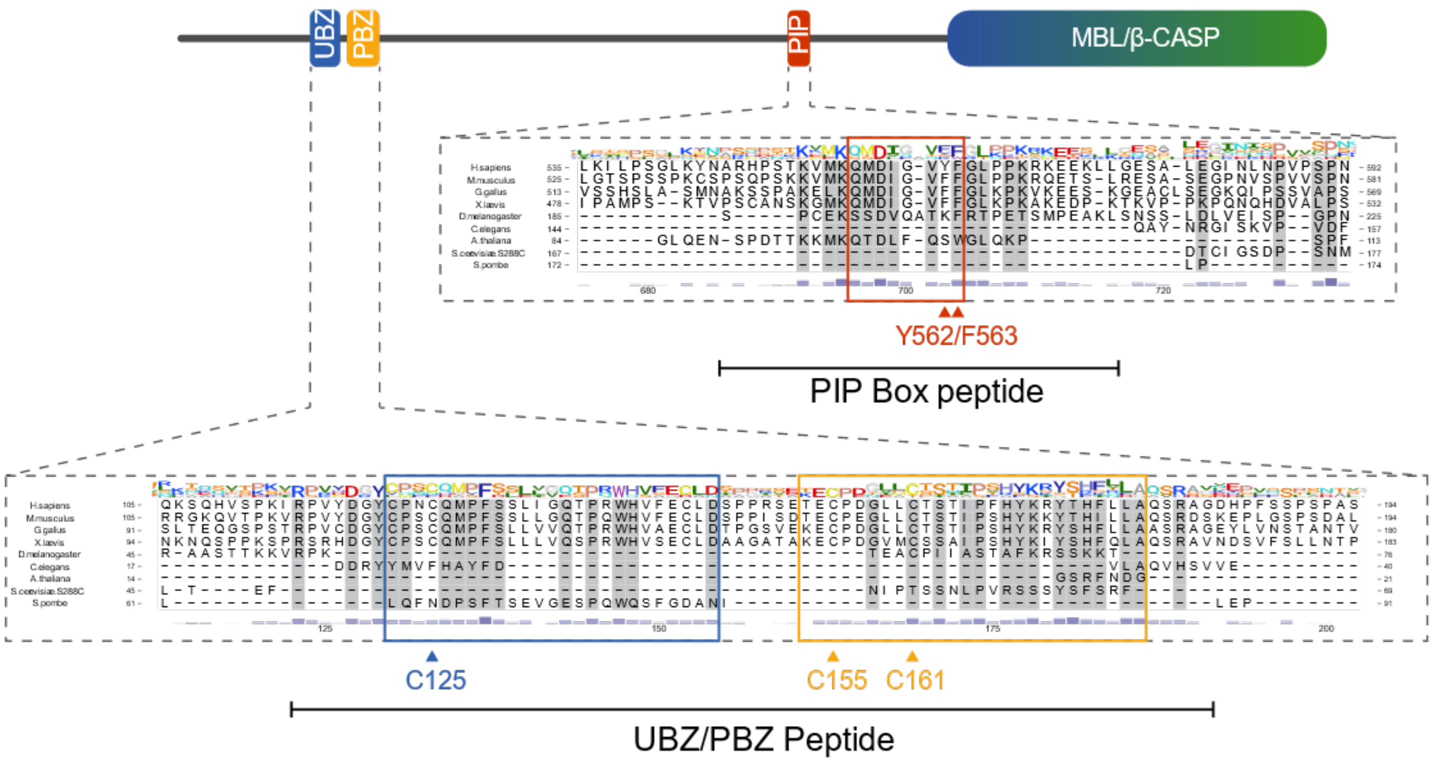


**Supplementary Figure 9** *Conserved domains and motifs of SNM1A.* Schematic of domains and motifs of the human SNM1A protein, with highlighted sequence alignments for the ubiquitin-binding zinc-finger 4 (UBZ), PAR-binding zinc-finger (PBZ) and PCNA-interacting peptide (PIP) box motifs. The mutated residues used in this study are highlighted, namely UBZ C125 (light blue), PBZ C155, C161 (yellow) and PIP box Y562, F563 (red), all which show strong conservation across vertebrates. The GST-SNM1A peptides used in Figure 3 to analyse the PAR, PCNA and PCNA$^{ub}$ binding are also indicated.

A

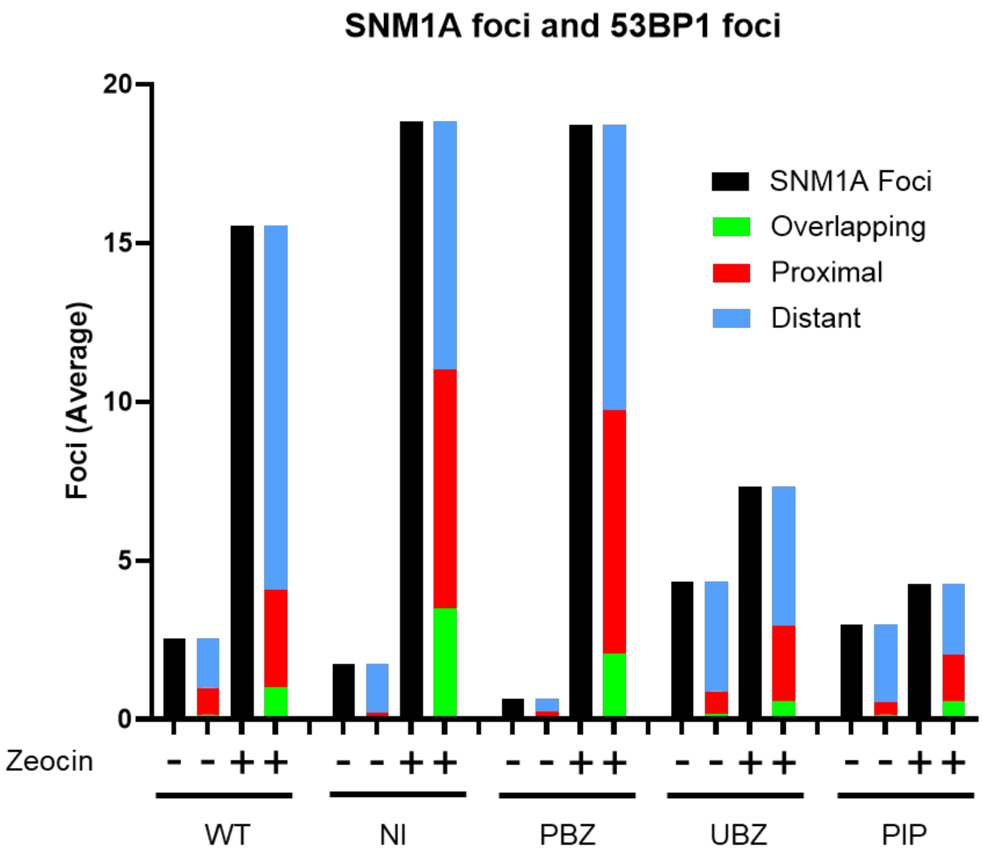


B

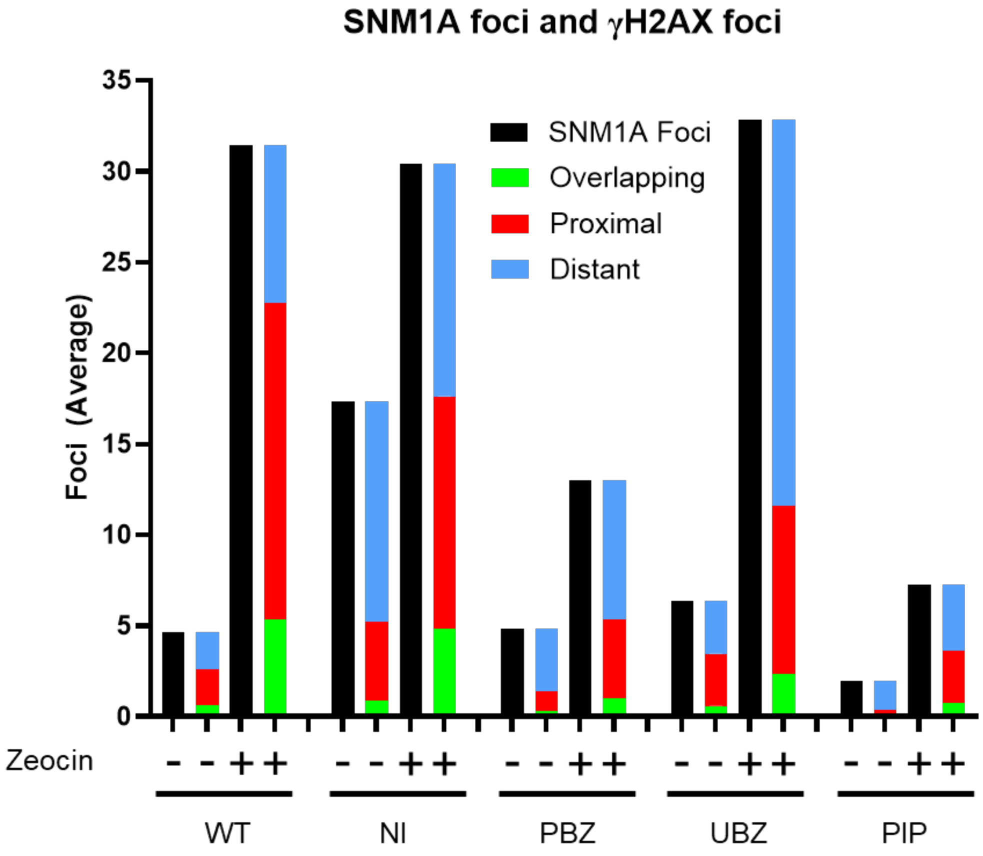


**Supplementary Figure 10** *Quantification of Zeocin-induced SNM1A foci and corresponding proximity of 53BP1 and γH2AX foci in wildtype and mutant transiently expressed EGFP-SNM1A.* Quantification of data

represented in **Figure 3.** Following Zeocin treatment (0.1 mg/mL, 2 hour) foci were measured for transient expression of EGFP-SNM1A WT, Nuclease inactive (NI), PBZ, UBZ and PIP box mutations, and the distance of these foci were compared to **A.** 53BP1 and **B.** H2AX foci. These were measured as overlapping (foci less than 0.36 µm, apart), proximal (0.36 to 1.07 µm, apart) and distant (greater than 1.07 µm apart) as outlined in the Methods section. Data is derived for **A** by counting (from left to right) 123, 497, 58, 93, 234, 82, 783, 277, 148, 146 cells, and for **B** by counting 25, 79, 138, 64, 67, 28, 109, 14, 23, 20 cells.

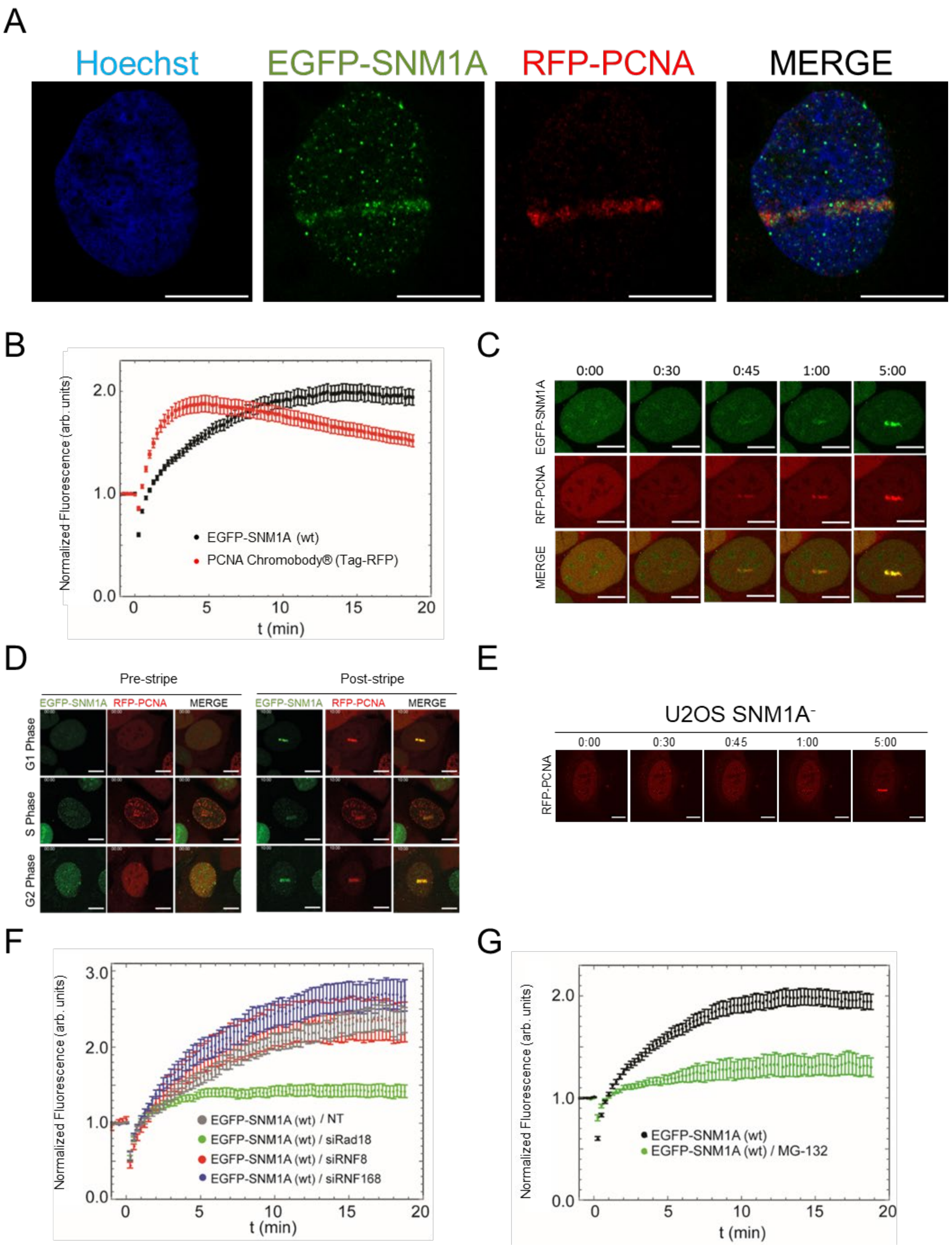

A
Hoechst
EGFP-SNM1A
RFP-PCNA
MERGE
B
Normalized Fluorescence (arb. units)
2.0
1.0
0.0
0
5
10
15
20
t (min)
EGFP-SNM1A (wt)
PCNA Chromobody® (Tag-RFP)
C
0:00
0:30
0:45
1:00
5:00
EGFP-SNM1A
RFP-PCNA
MERGE
D
Pre-stripe
Post-stripe
EGFP-SNM1A
RFP-PCNA
MERGE
G1 Phase
S Phase
G2 Phase
E
U2OS SNM1A-
0:00
0:30
0:45
1:00
5:00
RFP-PCNA
F
Normalized Fluorescence (arb. units)
3.0
2.0
1.0
0.0
0
5
10
15
20
t (min)
EGFP-SNM1A (wt) / NT
EGFP-SNM1A (wt) / siRad18
EGFP-SNM1A (wt) / siRNF8
EGFP-SNM1A (wt) / siRNF168
G
Normalized Fluorescence (arb. units)
2.0
1.0
0.0
0
5
10
15
20
t (min)
EGFP-SNM1A (wt)
EGFP-SNM1A (wt) / MG-132

**Supplementary Figure 11** *PCNA ubiquitination plays a role in SNM1A recruitment to DNA damage.* **A.** Cells stably expressing EGFP-SNM1A and an RFP-PCNA Chromobody® (Tag-RFP) were subject to laser-induced DNA damage, where co-localisation of EGFP-SNM1A (green) and PCNA (red) to the site of laser-induced DNA damage was observed. DNA was stained with Hoechst 333258 (blue) **B.** the rate and amount of fluorescence for EGFP-SNM1A and RFP-PCNA were plotted showing that the recruitment of PCNA precedes EGFP-SNM1A. **C.** 'snap shots' of recruitment of EGFP-SNM1A and RFP-PCNA to laser induced damage over the first 5 minutes following laser damage and **D.** representative snapshots of recruitment of EGFP-SNM1A and RFP-PCNA in different phases of the cell cycle (note: this is an extended figure to data presented in **Figure 4E**). **E.** RFP-PCNA recruitment in SNM1A$^{-}$ cells. **F.** Effect of siRNA depletion of the major E3 ligases involved in DSB repair, Rad18, RNF8 and RNF168 on the recruitment of EGFP-SNM1A to sites of laser damage. **G.** The effects of treatment with the proteasome inhibitor MG-132 (5 μM, 90 minutes) on EGFP-SNM1A recruitment to laser damage. All laser striping experiments represent data from at least eight cells from three biological repeats, error bars are standard error of the mean. Scale bars in A and C are 10 μm.

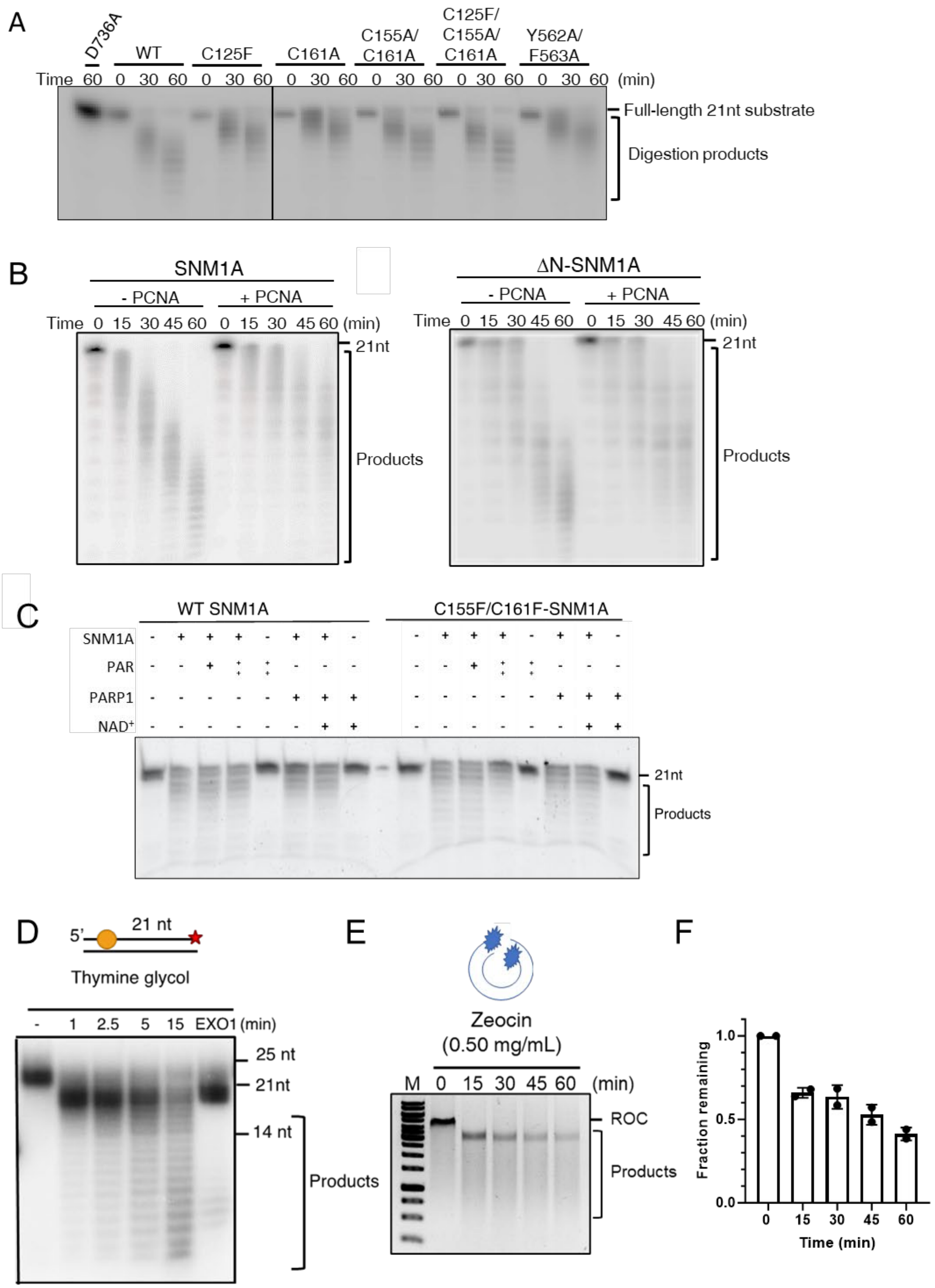
A
D736A
WT
C125F
C161A
C155A/ C161A
C125F/ C155A/ C161A
Y562A/ F563A
Time 60 0 30 60 0 30 60 0 30 60 0 30 60 0 30 60 0 30 60 (min)
Full-length 21nt substrate
Digestion products
B
SNM1A
- PCNA
+ PCNA
Time 0 15 30 45 60 0 15 30 45 60 (min)
21nt
Products
ΔN-SNM1A
- PCNA
+ PCNA
Time 0 15 30 45 60 0 15 30 45 60 (min)
21nt
Products
C
WT SNM1A
C155F/C161F-SNM1A
SNM1A
PAR
PARP1
NAD+
21nt
Products
D
5'
21 nt
Thymine glycol
- 1 2.5 5 15 EXO1 (min)
25 nt
21nt
14 nt
Products
E
Zeocin (0.50 mg/mL)
M 0 15 30 45 60 (min)
ROC
Products
F
Fraction remaining
1.0
0.5
0.0
0 15 30 45 60
Time (min)

**Supplementary Figure 12** *Mutations in SNM1A damage recruitment domains (UBZ, PBZ, PIP box) do not alter the enzymatic activity of SNM1A and neither PCNA nor PAR chains impact on SNM1A biochemical activity.* **A.** The digestion of 21-mer ssDNA oligonucleotide by full-length SNM1A, and SNM1A bearing mutations in the UBZ (C125F), PBZ (C161A, C155A), and PIP Box (Y562A, F563A), and combinations of these. **B.** Effect of PCNA on ssDNA digestion characteristics employing full-length SNM1A, or a truncated form consisting of the MBL-β-CASP fold catalytic domain of SNM1A (ΔN–SNM1A; residues 697-1040). **C.** Effect of poly-ADP-ribose chains (PAR) directly added to the reaction mix on the digestion of a 21-mer ssDNA oligonucleotide by SNM1A. Also shown is the effect of coincubation of PARP1 with $NAD^+$ to produce auto-PARylated PARP1, and the effect of this reaction on SNM1A activity. Reaction time was 30 minutes. **D.** SNM1A can digest a dsDNA substrate containing a thymine glycol lesion while EXO1 can not. **E.** Zeocin treated (0.5 mg/mL) pG46 plasmid DNA was incubated with purified SNM1A showing digestion over time. **F.** Quantification of remaining plasmid DNA as shown in **E.** normalised to T=0 (n=2, error = SD).
