# Supplementary material for: SNM1A is crucial for efficient repair of complex DNA breaks in human cells
Source: Nat Commun. 2024 Jun 25;15:5392. doi: 10.1038/s41467-024-49583-5 (PMC11199599; doi:10.1038/s41467-024-49583-5)
Supplement: Supplementary file 3 — Description of Additional Supplementary Files [file 41467_2024_49583_MOESM3_ESM.pdf]

Description of Additional Supplementary Files

File Name: Supplementary Movie 1

Description: Movie of recruitment of EGFP-SNM1A, its mutant forms and RFP-PCNA on to laser-induced DNA damage for wild-type EGFP-SNM1A, as well as combinations of mutants in the UBZ, PBZ and PIP box.

File Name: Supplementary Movie 2

Description: Representative movie of EGFP-SNM1A and RFP-PCNA recruitment to sites of laser-induced damage.
