# Supplementary material for: SNM1A is crucial for efficient repair of complex DNA breaks in human cells
Source: Nat Commun. 2024 Jun 25;15:5392. doi: 10.1038/s41467-024-49583-5 (PMC11199599; doi:10.1038/s41467-024-49583-5)
Supplement: Supplementary file 6 — Reporting Summary [file 41467_2024_49583_MOESM6_ESM.pdf]

nature portfolio

Corresponding author(s): Peter J. McHugh

Last updated by author(s): May 17, 2024

Reporting Summary

Nature Portfolio wishes to improve the reproducibility of the work that we publish. This form provides structure for consistency and transparency in reporting. For further information on Nature Portfolio policies, see our Editorial Policies and the Editorial Policy Checklist.

Statistics

For all statistical analyses, confirm that the following items are present in the figure legend, table legend, main text, or Methods section.

n/a	Confirmed	
☐	☒	The exact sample size (*n*) for each experimental group/condition, given as a discrete number and unit of measurement
☐	☒	A statement on whether measurements were taken from distinct samples or whether the same sample was measured repeatedly
☐	☒	The statistical test(s) used AND whether they are one- or two-sided<br>*Only common tests should be described solely by name; describe more complex techniques in the Methods section.*
☐	☒	A description of all covariates tested
☐	☒	A description of any assumptions or corrections, such as tests of normality and adjustment for multiple comparisons
☐	☒	A full description of the statistical parameters including central tendency (e.g. means) or other basic estimates (e.g. regression coefficient) AND variation (e.g. standard deviation) or associated estimates of uncertainty (e.g. confidence intervals)
☒	☐	For null hypothesis testing, the test statistic (e.g. *F*, *t*, *r*) with confidence intervals, effect sizes, degrees of freedom and *P* value noted<br>*Give P values as exact values whenever suitable.*
☒	☐	For Bayesian analysis, information on the choice of priors and Markov chain Monte Carlo settings
☒	☐	For hierarchical and complex designs, identification of the appropriate level for tests and full reporting of outcomes
☒	☐	Estimates of effect sizes (e.g. Cohen's *d*, Pearson's *r*), indicating how they were calculated

Our web collection on statistics for biologists contains articles on many of the points above.

Software and code

Policy information about availability of computer code

Data collection	ZEISS LSM 880<br>Bio-Rad ChemiDoc Imaging System<br>Vilber Fusion FX7 chemidoc system<br>BD FACSAria IIIu<br>Attune NxT flow cytometer<br>COLCount Colony counter, Oxford Optronix<br>GE Typhoon FLA 9500 imager
Data analysis	Graphpad Prism V9 and V10<br>Image J (Fiji) with custom macro script for counting foci (as deposited https://gist.github.com/dwaithe/ca774de63fdaae5a8e65a1d0059d61dc)<br>ColabFold Google Colabs notebook to predict structures<br>AlphaFold to create structural models<br>ChimeraX to render structural models<br>FCS Express V7<br>Mathematica

For manuscripts utilizing custom algorithms or software that are central to the research but not yet described in published literature, software must be made available to editors and reviewers. We strongly encourage code deposition in a community repository (e.g. GitHub). See the Nature Portfolio guidelines for submitting code & software for further information.

Data

Policy information about availability of data

All manuscripts must include a data availability statement. This statement should provide the following information, where applicable:

- Accession codes, unique identifiers, or web links for publicly available datasets
- A description of any restrictions on data availability
- For clinical datasets or third party data, please ensure that the statement adheres to our policy

Source data is provided with this paper

Research involving human participants, their data, or biological material

Policy information about studies with human participants or human data. See also policy information about sex, gender (identity/presentation), and sexual orientation and race, ethnicity and racism.

Reporting on sex and gender	*Use the terms sex (biological attribute) and gender (shaped by social and cultural circumstances) carefully in order to avoid confusing both terms. Indicate if findings apply to only one sex or gender; describe whether sex and gender were considered in study design; whether sex and/or gender was determined based on self-reporting or assigned and methods used. Provide in the source data disaggregated sex and gender data, where this information has been collected, and if consent has been obtained for sharing of individual-level data; provide overall numbers in this Reporting Summary. Please state if this information has not been collected. Report sex- and gender-based analyses where performed, justify reasons for lack of sex- and gender-based analysis.*
Reporting on race, ethnicity, or other socially relevant groupings	*Please specify the socially constructed or socially relevant categorization variable(s) used in your manuscript and explain why they were used. Please note that such variables should not be used as proxies for other socially constructed/relevant variables (for example, race or ethnicity should not be used as a proxy for socioeconomic status). Provide clear definitions of the relevant terms used, how they were provided (by the participants/respondents, the researchers, or third parties), and the method(s) used to classify people into the different categories (e.g. self-report, census or administrative data, social media data, etc.) Please provide details about how you controlled for confounding variables in your analyses.*
Population characteristics	*Describe the covariate-relevant population characteristics of the human research participants (e.g. age, genotypic information, past and current diagnosis and treatment categories). If you filled out the behavioural & social sciences study design questions and have nothing to add here, write "See above."*
Recruitment	*Describe how participants were recruited. Outline any potential self-selection bias or other biases that may be present and how these are likely to impact results.*
Ethics oversight	*Identify the organization(s) that approved the study protocol.*

Note that full information on the approval of the study protocol must also be provided in the manuscript.

Field-specific reporting

Please select the one below that is the best fit for your research. If you are not sure, read the appropriate sections before making your selection.

☒ Life sciences ☐ Behavioural & social sciences ☐ Ecological, evolutionary & environmental sciences

For a reference copy of the document with all sections, see nature.com/documents/nr-reporting-summary-flat.pdf

Life sciences study design

All studies must disclose on these points even when the disclosure is negative.

Sample size	Sample sizes were determined based on experience and following literature examples
Data exclusions	No data were excluded from the analysis
Replication	Cell-based experiments were replicated to include a minimum of three biological repeats unless otherwise staed. The number of repeats are stated in the figure legends and the statistical analysis for each experiment is also included in the figure legends.
Randomization	no randomization was required
Blinding	As data was analyzed with computer based programs, no blinding was required.

Reporting for specific materials, systems and methods

We require information from authors about some types of materials, experimental systems and methods used in many studies. Here, indicate whether each material, system or method listed is relevant to your study. If you are not sure if a list item applies to your research, read the appropriate section before selecting a response.

Materials & experimental systems

n/a	Involved in the study	
☐	☒	Antibodies
☐	☒	Eukaryotic cell lines
☒	☐	Palaeontology and archaeology
☒	☐	Animals and other organisms
☒	☐	Clinical data
☒	☐	Dual use research of concern
☒	☐	Plants

Methods

n/a	Involved in the study	
☒	☐	ChIP-seq
☐	☒	Flow cytometry
☒	☐	MRI-based neuroimaging

Antibodies

Antibodies used

Antibody Supplier Cat #(Clone#) Lot Number
Glutathione-S-Transferase Merck G1160 087H4806
APLF Abcam AB105446 GR119254-1
Anti-poly-ADP-ribose R&D systems 4335-MC-100 20554
SNM1A Bethyl Laboratories A303-747A 1
Ub PCNA (K164) Cell Signalling 134395 [D5C7P] 4
PCNA (Ms) Santa Cruz SC-056 [PC-10] C2922
PCNA (Rb) Abcam AB92552 [EPR3821] GR244165-29
BrdU Abcam AB6326 [BU1/75 (ICR1)] GR267766-1
β-Actin-HRP Merck A3854 [AC-15] 239228
53BP1 (Ms) Millipore MAB3802 [BP13] 3173790
53BP1 (Rb) Abcam AB175933 [EPR2172(2)] GR3225663-2
γH2AX (Ms) Millipore 05-636-I [JBW301] 3308857
γH2AX (Rb) Abcam Ab81299 [EP854(2)Y] GR3203642-12
Goat anti-Rabbit-HRP Dako P0448 41424306
Goat anti-Mouse-HRP Dako P0447 000776656
Alexa Fluor 555 (anti-Rabbit) Invitrogen A31572 5294873
Alexa Fluor 647 (anti-Rabbit) Invitrogen A21443 1917946
Alexa Fluor 555 (anti-Mouse) Invitrogen A31570 1575605
Alexa Fluor 647 (anti-Mouse) Invitrogen A31571 1839633
HA.11 BioLegend 901533 [16B12] B350093 Mouse Mono

Validation

Antibodies were validated by ourselves, or by the manufacturers. We used a variety of methods depending on the protein product including siRNA knockdown coupled with SDS PAGE/western blot analysis, Q-RT-PCR, purified protein binding.

Eukaryotic cell lines

Policy information about cell lines and Sex and Gender in Research

Cell line source(s)

U2OS (purchased through American Type Culture collection: HTB-96), 293FT (purchased from ThermoFisher Scientific: R70007), HEK293 XRCC4- (a gift from Andrew Blackford), 293T PCNAK164R cells (a gift of George-Lucian Moldovan).

Authentication

U2OS and U2OS/SNM1A- were authenticated by LGC standards.

Mycoplasma contamination

Cell lines were routinely tested for mycoplasma using Mycoalert kit from Lonza. Cell used in this study were mycoplasma negative

Commonly misidentified lines
(See ICLAC register)

Name any commonly misidentified cell lines used in the study and provide a rationale for their use.

Flow Cytometry

Plots

Confirm that:

☐ The axis labels state the marker and fluorochrome used (e.g. CD4-FITC).

☐ The axis scales are clearly visible. Include numbers along axes only for bottom left plot of group (a 'group' is an analysis of identical markers).

☐ All plots are contour plots with outliers or pseudocolor plots.

☐ A numerical value for number of cells or percentage (with statistics) is provided.

Methodology

Sample preparation	cells in culture were either sorted for fluorescent positive cells, or analyzed for DNA content as per method chapter.
Instrument	Cell sorting was performed on FACSAria IIIu, Flow cytometry was performed on Attune NxT instruments.
Software	FCS Express V7 was used to analyze the data
Cell population abundance	For sorting, fluorescent populations were usually between 5-15%.
Gating strategy	Forward versus side scatter was used (against control populations) to establish the viable populations. Doublets were removed by plotting forward scatter height versus area. See example in Suppl Fig 3a

☒ Tick this box to confirm that a figure exemplifying the gating strategy is provided in the Supplementary Information.
